# Transgenic *fat-1 *mouse as a model to study the pathophysiology of cardiovascular, neurological and psychiatric disorders

**DOI:** 10.1186/1476-511X-8-61

**Published:** 2009-12-30

**Authors:** Undurti N Das, László G Puskás

**Affiliations:** 1 Jawaharlal Nehru Technological University, Kakinada-533 003, Andhra Pradesh, India; 2 Functional Genomics Laboratory, Biological Research Center of the Hungarian Academy of Sciences, Temesvári krt 62, Szeged H-6726, Hungary; 3 UND Life Sciences, 13800 Fairhill Road, #321, Shaker Heights, OH 44120, USA

## Abstract

Polyunsaturated fatty acids (PUFAs) form an important constituent of all the cell membranes in the body. PUFAs such as arachidonic acid (AA), eicosapentaenoic acid (EPA) and docosahexaenoic acid (DHA) form precursors to both pro-inflammatory and anti-inflammatory compounds. Low-grade systemic inflammation occurs in clinical conditions such as insulin resistance, hypertension, type 2 diabetes mellitus, atherosclerosis, coronary heart disease, lupus, schizophrenia, Alzheimer's disease, and other dementias, cancer and non-alcoholic fatty liver disease (NAFLD) that are also characterized by an alteration in the metabolism of essential fatty acids in the form of excess production of pro-inflammatory eicosanoids and possibly, decreased synthesis and release of anti-inflammatory lipoxins, resolvins, protectins and maresins. We propose that low-grade systemic inflammation observed in these clinical conditions is due to an imbalance in the metabolism of essential fatty acids that is more in favour of pro-inflammatory molecules. In this context, transgenic *fat-1 *mouse that is designed to convert n-6 to n-3 fatty acids could form an ideal model to study the altered metabolism of essential fatty acids in the above mentioned conditions. It is envisaged that low-grade systemic inflammatory conditions are much less likely in the *fat-1 *mouse and/or these diseases will run a relatively mild course. Identifying the anti-inflammatory compounds from n-3 fatty acids that suppress low-grade systemic inflammatory conditions and understanding their mechanism(s) of action may lead to newer therapeutic strategies.

## Introduction

One traditional approach to modify tissue nutrient composition to study the effects of different diets is by supplementing the experimental groups with different diets consisting of many variations. Although this is an accepted mode of studying the effect of various nutrients and their effects on various physiological processes and pathologic situations, it is difficult to make all the dietary components identical, except the total energy, between two diets and perhaps the single component in which one is interested. The inevitable differences between diets and their components, however small they may be, may confound the study and contribute to inconsistencies or conflicting results observed. This is especially so for studies when one wishes to know the specific actions and functions of ω-3 and ω-6 fatty acids. In general, the effects of ω-3 and ω-6 fatty acids are studied supplementing the experimental groups of animals with different ω-3/ω-6 fatty acid ratios to establish the different fatty acid profiles in the tissues and extrapolate the results so obtained to the observed differences in the indices examined. In these studies, generally, fish oils and plant seed/vegetable oils are used to provide the required ω-3/ω-6 fatty acids respectively. Since these fatty acids are derived from different sources and are likely to contain other bioactive compounds, however minor they might be, are likely to affect the study outcomes. Furthermore, polyunsaturated fatty acids (PUFAs) are highly unstable and susceptible to oxidation. These variables arising from the diet and feeding procedures invariably affect the results.

### *Fat-1 *mouse

**Figure 1:**
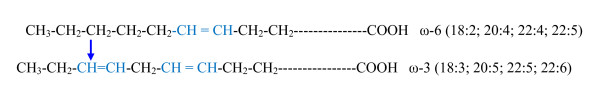
**The *fat-1 *gene encodes an n-3 desaturase that converts n-6 to n-3 fatty acid**.

In view of these issues, it is ideal to develop a transgenic mouse capable of converting ω-6 to ω-3 fatty acids so that the results obtained in such an animal model will be more reliable and easy to interpret in terms of the effects of ω-3 and ω-6 fatty acids. Mice engineered to carry *fat-1 *gene from *Caenorhabditis elegans *can add a double bond into an unsaturated fatty acid hydrocarbon chain and convert ω-6 to ω-3 fatty acids as shown in Figure 1. This resulted in an abundance of ω-3 and a reduction in ω-6 fatty acids in the organs and tissues of these mice even in the absence of dietary ω-3 fatty acids [1]. Thus, *fat-1 *mouse model offers an opportunity for investigating the biological functions of ω-3 fatty acids and the importance of the ratio of ω-3/ω-6 in various physiological processes and diseases. When both transgenic and wild type mice were maintained on a diet rich in ω-6 fatty acids (mainly linoleic acid, 18:2) with very little ω-3 fatty acids (~ 0.1% of total fat supplied), wild type mice showed little or no ω-3 fatty acids in their tissues because they cannot produce ω-3 from ω-6 fatty acids, while the *fat-1 *transgenic mice showed significant amounts of ω-3 fatty acids in their tissues (1, and see table 1 for differences in the ratio between ω-3 and ω-6 fatty acids in various tissues between wild-type and fat-1 mice). The transgenic mice were found to be normal and healthy and many generations of transgenic mouse lines have been examined and their tissue fatty acid profiles showed consistently high levels of ω-3 fatty acids, indicating that the transgene is transmittable.

**Table 1:** Fatty acid ratio (ω-6/ω-3) in wild-type and *fat-1 *mice.

	ω-6/ω-3 ratio	
**Tissue**	**Wild-type**	***fat-1***

Muscle	49.0	0.7
Milk	32.7	5.7
RBC	46.6	2.9
Heart	22.8	1.8
Brain	3.9	0.8
Liver	26.0	2.5
Kidney	16.5	1.7
Lung	32.3	2.2
Spleen	23.8	2.4

Preliminary studies have already confirmed that transgenic *fat-1 *mouse is resistant to colitis [2], chemical-induced hepatitis due to dampening of inflammatory reaction [3] and post-ovariectomy osteoporosis [4]. Colitis, chemical-induced hepatitis and osteoporosis are all due to enhanced production of cytokines: inerleukin-6 (IL-6) and tumor necrosis factor-α (TNF-α) and since these diseases are much less severe in transgenic *fat-1 *mouse indicates that presence of enhanced amounts of ω-3 fatty acids in the respective tissues and plasma has a dampening effect on inflammation. In all these studies except in the colitis model study [2], plasma and tissue levels of ω-3 fatty acid products lipoxins, resolvins, protectins and maresins were not estimated.

A striking reduction in melanoma development and progression in *fat-1 *transgenic mice in parallel with an increase in the levels of PGE_3 _(prostaglandinE_3_) derived from EPA and higher ω-3/ω-6 ratio in the tumor and surrounding tissue of *fat-1 *mice compared to wild type animal was reported [5]. The PTEN (a tumor suppressor) gene was significantly up-regulated in the *fat-1 *mice. We propose that the beneficial actions seen in transgenic *fat-1 *mouse with regard to less severe colitis, chemical-induced hepatitis, osteoporosis and anti-cancer activity are all due to enhanced formation of lipoxins, resolvins, protectins and maresins derived from ω-3 EPA and DHA. This implies that lipoxins, resolvins, protectins and maresins have anti-atheroslcerotic, cardioprotective actions and anti-cancer actions, especially following acute myocardial infarction and ischemia-reperfusion injury.

Despite these benefits noted with the *fat-1 *transgenic mice, it is not without side effects. Ji et al [6] developed seven lines of *fat-1 *transgenic mice (C57/BL6) controlled by the regulatory sequences of the adipocyte protein-2 (aP2) gene for adipocyte-specific expression (AP-lines). Ji et al [6] were unable to obtain homozygous *fat-1 *transgenic offspring from the two highest expressing lines, suggesting that excessive expression of this enzyme may be lethal during gestation. As expected, it was noted that serum fatty acid analysis of *fat-1 *transgenic mice (AP-3) fed a high ω-6 unsaturated fat diet had a ω-6/ω-3 fatty acid ratio reduced by 23% and the ω-3 fatty acid eicosapentaenoic acid (EPA) concentration increased by 61%. Docosahexaenoic acid (DHA) was increased by 19% in white adipose tissue. Male AP-3-fat-1 line of mice had improved glucose tolerance and reduced body weight with no change in insulin sensitivity when challenged with a high-carbohydrate diet, while the female AP-3 mice had reduced glucose tolerance and no change in insulin sensitivity or body weight. These findings indicate that male transgenic *fat-1 *mice have improved glucose tolerance likely due to increased insulin secretion while female *fat-1 *mice have reduced glucose tolerance compared to wild-type mice. The inability of *fat-1 *transgenic mice to generate homozygous offspring suggests that prolonged exposure to increased concentrations of ω-3 fatty acids may be detrimental to reproduction [6].

Since some of the beneficial actions of PUFAs are attributed to the formation of anti-inflammatory molecules derived from AA, EPA and DHA, it is pertinent to discuss briefly here the metabolism of essential fatty acids.

### Metabolism of essential fatty acids

**Figure 2:**
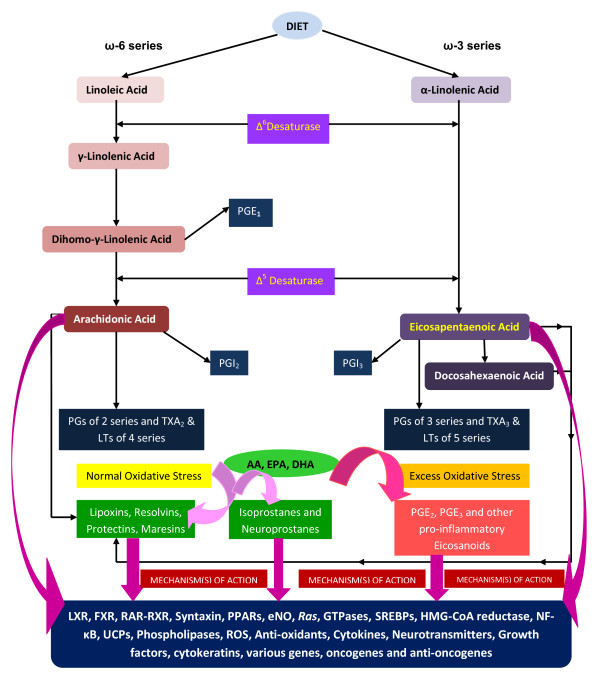
**Scheme showing the metabolism of essential fatty acids**.

Essential fatty acids (EFAs) are important constituents of all cell membranes and confer on membranes properties of fluidity and thus, determine and influence the behaviour of membrane-bound enzymes and receptors. EFAs are essential and cannot be synthesized in the body and hence, have to be obtained in our diet [7]. There are two types of EFAs, the ω-6 series derived from *cis*-linoleic acid (LA, 18:2) and the ω-3 series derived from α-linolenic acid (ALA, 18:3). LA is converted to γ-linolenic acid (GLA, 18:3, n-6) by the action of the enzyme Δ^6 ^desaturase (Δ^6^d) and GLA is elongated to form dihomo-GLA (DGLA, 20:3, n-6), the precursor of the 1 series of prostaglandins (PGs). DGLA can also be converted to arachidonic acid (AA, 20:4, n-6) by the action of the enzyme Δ^5 ^desaturase (Δ^5 ^d). AA forms the precursor of 2 series of prostaglandins, thromboxanes and the 4 series of leukotrienes. ALA is converted to eicosapentaenoic acid (EPA, 20:5, n-3) by Δ^6 ^and Δ^5^desaturases. EPA forms the precursor of the 3 series of prostaglandins and the 5 series of leukotrienes. LA, GLA, DGLA, AA, ALA, EPA and docosahexaenoic acid (DHA, 22:6, n-3) are all PUFAs, but only LA and ALA are EFAs (see Figure 2 for metabolism of EFAs). AA and EPA also give rise to their respective hydroxy acids, which in turn are converted to their respective leukotrienes (LTs). Both PGs and LTs are highly biologically active and have pro-inflammatory action, and are known to be involved various pathological processes. Many of the functions of EFAs are also brought about by PUFAs and EFA-deficiency states can be corrected to a large extent by PUFAs.

Studies revealed that PUFAs themselves play a significant role in the pathobiology of clinical conditions. This is in addition to the role of PGs and LTs in these conditions. For instance, in inflammatory bowel disease the inflammatory events seem to be initiated and perpetuated by PGs and LTs (such as PGE_2_, PGF_2α_, TXA_2 _and LTB_4_, LTC_4_, and LTD_4_) produced from AA, whereas when significant amounts of EPA and DHA are given the inflammatory process is abrogated to a large extent. This beneficial action of EPA/DHA when supplemented from external sources has been attributed to the displacement AA from the cell membrane phospholipid pool and to the formation of less pro-inflammatory PGs (such as PGE_3_, PGF_3α_, TXA_3_), and LTs (such as LTB_5_, LTC_5_, and LTD_5_) from them and hence the favorable response (see Figure 2 for metabolism of EFAs and various products formed from them).

AA, EPA and DHA also give rise to anti-inflammatory molecules such as lipoxins (LXs), resolvins, protectins and maresins and nitrolipids. LXs, resolvins, protectins and maresins (see Figure 2 for metabolism of essential fatty acids and the formation of resolvins, protectins, lipoxins and maresins from PUFAs) suppress inflammation and help in the resolution of inflammatory events including leukocyte infiltration and clearance of the cellular debris from the site of inflammation. This suggests that PUFAs form precursors to both pro- and anti-inflammatory molecules and the balance between these mutually antagonistic compounds could determine the final outcome of the disease process that forms the basis of generating *fat-1 *mice and favorable results seen with *fat-1 *mice [1-6].

EFAs and PUFAs play a significant role in various diseases and especially in cardiovascular and neurological/psychiatric disorders [8-15]. This is in addition to the role of PGs and LTs in these conditions. AA, EPA and DHA give rise to anti-inflammatory molecules lipoxins (LXs), resolvins, protectins and maresins that suppress inflammation. Thus, PUFAs form precursors to both pro- and anti-inflammatory molecules and the balance between these mutually antagonistic compounds could determine the final outcome of the disease process. Biologically active compounds formed due to the nitration of unsaturated fatty acids called as nitrolipids have also been identified. Nitrolipids stimulate smooth muscle relaxation, block platelet activation, inhibit human neutrophil functions and suppresses inflammation. Thus, PUFAs have many important actions not only by themselves but also by giving raise to various biologically active compounds.

### ω-6 and ω-3 balance and their role in cardiovascular, neurological and psychiatric disorders

It has been suggested that a substantial fall in the intake of ω-3 fatty acids could be one of the major changes in Western nutrition in the last 50 years that contributed to the increasing incidence of cardiovascular and neurological/psychiatric disorders. By increasing the intake of either ω-6 or ω-3 fatty acids, the plasma and tissue content of these fatty acids could be enhanced. In general, an increase in the content of ω-6 fatty acids such as AA is expected to lead an increase in the formation of pro-inflammatory PGs, LTs and TXs; whereas an increase in the content of ω-3 fatty acids such as EPA and DHA could lead to enhanced formation of less pro-inflammatory compounds such as PGI_3_, TXA_3_, LTs of 5 series and anti-inflammatory compounds lipoxins, resolvins, protectins and maresins that can prevent or even resolve the development of atherosclerosis, coronary heart disease, hypertension, Alzheimer's disease, schizophrenia, depression, Huntington's disease and other cardiovascular and neurological diseases that are associated with inflammatory events. In view of this, it has been recommended that both normal individuals and subjects who are at high risk of developing these diseases and patients enhance their intake of ω-3 fatty acids especially EPA and DHA.

Mammals cannot convert ω-6 to ω-3 fatty acids since they lack the gene for this purpose. Using transgenic approach, Kang et al (1) heterologously expressed the *C. elegans fat-1 *gene in mice. The resultant mice not only showed enhanced concentrations of ω-3 ALA, EPA, DHA, and docosapentaenoic acid (DPA), but also significantly reduced ω-6 LA and AA in muscle, red blood cells, heart, brain, liver, kidney, lung, and spleen tissues such that the ratio of ω-6 to ω-3 dropped from 20-50 to almost 1. Despite such a drastic change in the ratio of ω-6 to ω-3 the transgenic mice were found to be normal and healthy though they showed inability to generate homozygous offspring [6]. The *fat-1 *transgenic mouse model is ideal to study the effects of tissue ω-6/ω-3 ratio in the body and to delineate the exact molecular mechanism of actions of ω-3 fatty acids and their metabolites.

### ω-6 and ω-3 imbalance in cardiovascular diseases

EFAs and their long-chain metabolites such as GLA, DGLA, AA, EPA and DHA and other products such as prostaglandins E_1 _(PGE_1_), prostacyclin (PGI_2_), PGI_3_, lipoxins (LXs), resolvins, protectins and maresins prevent platelet aggregation, lower blood pressure, have anti-arrhythmic action, reduce LDL-C, ameliorate the adverse actions of homocysteine, show anti-inflammatory actions, activate telomerase, and have cytoprotective properties. Of all the PUFAs: DHA = EPA > GLA > DGLA when their actions on platelet aggregation, ability to lower blood pressure, prevent cardiac arrhythmias, reduce LDL-C, ameliorate the adverse actions of homocysteine, anti-inflammatory action, activation of telomerase and cytoprotective properties are compared. On the other hand, AA may enhance the production of pro-inflammatory eicosanoids and thus, could be harmful though it should be noted that anti-inflammatory compounds such as lipoxins are also formed from AA. Hence, the biological actions of AA in the body seem to be rather tricky. Under certain circumstance, AA may actually be beneficial by giving rise to lipoxins whereas at times it may be harmful by increasing the production of pro-inflammatory eicosanoids. Several studies showed that in patients with CHD, hypertension and type 1 and type 2 diabetes mellitus, plasma and tissue levels of AA have been shown to be low (reviewed in [15-17]) suggesting that perhaps in these conditions enhancing the tissue and plasma levels of AA could be beneficial. It has not been shown but, it is possible that when plasma and tissue levels of AA are normal the formation of lipoxins could be optimum such that inappropriate platelet aggregation, atherosclerosis and inflammation would not occur. Thus, under physiological conditions EFAs and their metabolites show all the classic actions expected of the "polypill" [17]. Furthermore, EFAs are endogenous molecules present in almost all tissues, have no significant or few side effects, can be taken orally for long periods of time even by pregnant women, lactating mothers, and infants, children, and adults; and reduce the incidence of cardiovascular diseases including stroke. In addition, various EFAs and their long-chain metabolites (including lipoxins, resolvins, protectins and maresins) not only enhance nitric oxide generation but also react with nitric oxide to yield their respective nitroalkene derivatives that produce vascular relaxation, inhibit neutrophil degranulation and superoxide formation, inhibit platelet activation, and possess PPAR-γ ligand activity and release NO, thus prevent platelet aggregation, thrombus formation, atherosclerosis, and cardiovascular diseases. These evidences suggest that a rational combination of ω-3 and ω-6 fatty acids and the co-factors that are necessary for their appropriate action/metabolism is as beneficial as that of the combined use of a statin, thiazide, a β blocker, and an angiotensin converting enzyme (ACE) inhibitor, folic acid, and aspirin. Furthermore, appropriate combination of ω-3 and ω-6 fatty acids may even show additional benefits in the form of protection from depression, schizophrenia, Alzheimer's disease, and enhances cognitive function; and serve as endogenous anti-inflammatory molecules; and could be administered from childhood for life long [17].

Based on these evidences, it is suggested that studies can be performed in transgenic *fat-1 *mouse to verify some of the above postulations. For instance, we hypothesize that transgenic *fat-1 *mouse will be resistant to atherosclerosis, thrombosis, CHD, stroke and inflammatory conditions such as inflammatory bowel diseases, collagen vascular diseases such as rheumatoid arthritis and lupus, ischemia-reperfusion injury, and cardiac arrhythmias following myocardial infarction.

Preliminary studies have already confirmed that transgenic *fat-1 *mouse is indeed resistant to colitis (2), chemical-induced hepatitis due to dampening of inflammatory reaction (3) and post-ovariectomy osteoporosis (4). Colitis, chemical-induced hepatitis and osteoporosis are due to enhanced production of pro-inflammatory cytokines. Since these diseases are much less severe in transgenic *fat-1 *mouse indicates that presence of enhanced amounts of ω-3 fatty acids in the respective tissues and plasma has a dampening effect on inflammation. In all these studies except in the colitis model study (2), plasma and tissue levels of ω-3 fatty acid products lipoxins, resolvins, protectins and maresins were not estimated. We propose that the beneficial actions seen in transgenic *fat-1 *mouse with regard to less severe colitis, chemical-induced hepatitis and osteoporosis are due to enhanced formation of lipoxins, resolvins, protectins and maresins derived from ω-3 EPA and DHA.

### ω-6 and ω-3 imbalance and schizophrenia, Huntington's disease, and Alzheimer's disease

Low-grade systemic inflammation plays a significant role in the pathobiology of schizophrenia, Huntington's disease, depression and Alzheimer's disease. In patients with schizophrenia, both circulating and cerebrospinal fluid (CSF) concentrations of pro-inflammatory cytokines are increased and the plasma phospholipid concentrations of EPA and DHA are decreased. Supplementation of EPA (especially ethyl EPA) was reported to be of some benefit to these patients (reviewed in [11-14]).

Diet high in DHA slowed the progression of Alzheimer's disease (AD) in mice. Specifically, DHA cut the harmful brain plaques that mark the disease. Mice genetically altered to develop Alzheimer's disease, when were fed with DHA-fortified chow had 70-percent less buildup of amyloid protein in the brain compared with control or DHA-deficient mice (14, 18-20). DHA protected against damage to the "synaptic" areas and enabled mice to perform better on memory tests.

An inverse relationship has been found between transthyretin CSF level, an amyloid plaque scavenger, and the severity of dementia in AD patients [21]. In response to diet having high concentration of ω-3 in old rat hippocampus the expression of the transthyretin gene was dramatically induced [22], suggesting a positive role of ω-3 in AD patients. On the other hand, our data on the perinatal influence of dietary ω-3 depletion and the expression of the ZnT3 in the brain (and subsequent sequestration of zinc into brain tissue) could provide an important link between the positive effects of dietary DHA and reduced brain zinc on the pathology of AD [23]. It would be interesting to study the brain zinc distribution in the *fat-1 *transgenic mouse brain.

These studies indicate that increased intake of DHA could be of benefit in people who are genetically or otherwise predisposed to develop the disease. Furthermore, recent studies [24] in *fat-1 *transgenic mice showed that increased brain DHA significantly enhances hippocampal neurogenesis as evidenced by an increase in the number of proliferating neurons and increased density of dendritic spines of CA1 pyramidal neurons in the hippocampus. Concurrently, *fat-1 *mice exhibited a better spatial learning performance in the Morris water maze compared with control WT (wild-type) littermates. In vitro experiments further demonstrates that DHA promotes differentiation and neurite outgrowth of neuronal cells derived from mouse ES cells and increases the proliferation of cells undergoing differentiation into neuronal lineages from the ES cells. These results provide direct evidence that DHA promotes neurogenesis and neuritogenesis and thus, ω-3 fatty acids are beneficial in the prevention and treatment of Alzheimer's disease and improve behavioral performance.

Huntington's disease is an inherited neurodegenerative disorder due to a mutation in exon 1 of the *Huntingtin *gene that encodes a stretch of polyglutamine (poly Q) residues close to the N-terminus of the *Huntingtin *protein. Aggregated poly Q residues are toxic to the neuronal cells. Transgenic R6/1 mice that develop late-onset neurologic deficits similar to the motor abnormalities of Huntington's disease seen in humans showed increased survival rates and decreased neurologic deficits when were supplemented with PUFAs, especially ethyl EPA [13], suggesting that unsaturated fatty acids may prevent or arrest poly Q aggregation. These results suggest that PUFAs, in general, are useful in the treatment of various neurological diseases. Some of the beneficial actions of these PUFAs in neurological diseases could be due to the increased formation of lipoxins and resolvins that have neuroprotective actions. But, it is not clear why and how a particular fatty acid is useful only in a specific neurological condition. For instance, DHA is useful in Alzheimer's disease whereas ethyl EPA is of benefit in Huntington's disease and schizophrenia. More research is needed to understand the molecular mechanisms of action of EPA/DHA in these neurological conditions.

These evidences suggest that increased intake of EPA/DHA may prevent Alzheimer's disease, schizophrenia and Huntington's disease. Even after the onset of the disease, supplementation of EPA/DHA appears to either prevent further progression or even reverse some of the features of these diseases. Since transgenic *fat-1 *mouse is able to convert ω-6 to ω-3 fatty acids and thus, contain significantly higher amounts of EPA/DHA in several tissues including brain, we hypothesize that Alzheimer's disease, schizophrenia, depression and Huntington's disease are unlikely to occur in this mouse model or even if they occur they will be of less severity. Hence, it is important to study in the transgenic *fat-1 *mouse model what metabolites of EPA/DHA are responsible for their protective action against Alzheimer's disease, schizophrenia, depression and Huntington's disease. It is highly likely that lipoxins, protectins, resolvins and maresins are formed in significantly higher amounts in the transgenic *fat-1 *mouse that accounts for the beneficial actions of EPA/DHA.

### Actions of EFAs and their metabolites that could account for their beneficial actions

#### Cell membrane fluidity

Cell membrane fluidity is determined by its lipid composition: increasing its content of saturated fatty acids and cholesterol renders the membrane more rigid, whereas increasing unsaturated fatty acids makes it more fluid. This is an important function of lipids since the number of receptors and their affinity to their respective hormones/growth factors/proteins depends on the fluidity of the cell membrane.

Availability of appropriate amounts of ω-3 and ω-6 fatty acids and various growth factors is essential for the growth of brain during the perinatal period and adolescence [7,11-13,24-26]. Deficiency of ω-3 EPA and DHA and ω-6 AA during the critical growth period impairs brain growth and the development of appropriate synaptic connections that, in turn, could lead to developmental disorders of the brain and neuropsychological conditions: dementia, depression, schizophrenia, Alzheimer's disease, and neurodegenerative diseases: Huntington's disease, Parkinson's disease, spinocerebellar degeneration, etc, and may impair memory formation and consolidation. We suggest that the fluidity of the cell membranes of the neurons and other cells in transgenic *fat-1 *mouse will be more fluid compared to wild type mouse. The increase in cell membrane fluidity will enable growth factors and neurotransmitters to bind to their respective receptors with high affinity that may protect transgenic *fat-1 *mouse against Alzheimer's disease, schizophrenia and Huntington's disease.

We found that combined application of cholesterol a compound that makes membranes more rigid and PUFA that increase the fluidity of biological membranes had different effects on inflammatory gene expressions in brain and in the eye [21-23]. We hypothesize that in the *fat-1 *transgenic mouse cholesterol has much less negative effects than in the wild-type mouse.

#### Endothelial nitric oxide generation

EPA/DHA enhances endothelial nitric oxide generation [27]. Plasma and tissue concentrations of PUFA and eNO are low in dementia, schizophrenia, bipolar disorders, Huntington's disease and Alzheimer's disease [7,11-13,18-20,27,28].

NO is a potent anti-atherosclerotic and anti-inflammatory molecule. Aspirin enhances the formation of eNO through the generation of epi-lipoxins that may explain its anti-inflammatory action [29]. Epi-lipoxins that have potent anti-inflammatory actions and enhance the generation of NO, whereas NO stimulates the formation of PGI_2 _from AA [30] and lipoxins are derived from AA, EPA, and DHA. These results emphasize the close interaction between PUFAs, NO synthase, and COX enzymes [31]. Furthermore, PUFAS can react with NO to form nitrolipids that can release NO. Based on these results, we propose that transgenic *fat-1 *mouse produce increased amounts of eNO, PGI_2 _and PGI_3 _and lipoxins compared to the wild-type that could also account for the decreased incidence of various cardiovascular and neurological and psychiatric disorders.

#### Suppression of production of pro-inflammatory cytokines

EPA, DHA, LXs, resolvins, protectins and maresins suppress pro-inflammatory IL-1, IL-2, IL-6, macrophage migration inhibitory factor (MIF), HMGB1 (high mobility group box 1) and TNF-α production by T cells and other cells (7, 32-35), and thus could function as endogenous anti-inflammatory molecules. PGE_2_, PGF_2α_, TXA_2 _and LTs derived from AA also modulate IL-6 and TNF-α production. These results imply levels of IL-6 and TNF-α at the sites of inflammation and injury may depend on the local levels of various PUFAs and eicosanoids formed from them. The ability of EPA and DHA to suppress the production of pro-inflammatory cytokines and induce their anti-inflammatory actions are mediated by their ability to increase PPAR-γ mRNA and protein activity [35].

IL-1, IL-6, MIF (macrophage migration inhibitory factor) and TNF-α induce insulin resistance, have cytotoxic actions, are neurotoxic, and seem to have a role in the pathobiology of cardiovascular and neurologic and psychiatric conditions. Hence, we predict that in transgenic *fat-1 *mouse the production of pro-inflammatory cytokines will be low compared to the wild type.

#### HMG-CoA reductase activity

Similar to statins, EPA and DHA are useful in the treatment of hyperlipidemias. More importantly, EPA and DHA are potent inhibitors of the HMG-CoA reductase enzyme [36,37]. Statins enhance plasma PUFA concentrations and decrease the ratio of EPA to AA significantly [38].

PUFAs down regulate hepatic cholesterol synthesis by impairing the SREBP (sterol regulatory element-binding protein) pathway; reduce SREBP-mediated gene transcription by increasing intracellular cholesterol content through the hydrolysis of cellular sphingomyelin; decrease SRE-mediated gene transcription of SREBP-1 and SREBP-2 and thus, PUFAs modulate the function of SREBPs [39-43].

HMG-CoA reductase catalyzes the synthesis of mevalonate, which is the rate-limiting step in the mevalonate pathway. Mevalonate is the precursor of cholesterol and a variety of isoprenoid containing compounds. These isoprenoid precursors are necessary for the posttranslational lipid modification (prenylation) and hence, the function of *Ras *and other small GTPases. Hence, inhibition of mevalonate pathway has the potential to disrupt the function of oncogenic forms of *Ras*. This explains the ability of PUFAs, especially EPA and DHA to suppress *Ras *activity, anti-proliferative action and induce apoptosis of tumor cells. Small GTPases, which are prenylated products of the mevalonate pathway, have negative control on the expression of BMPs (bone morphogenetic proteins). Thus, inhibition of the mevalonate pathway by EPA and DHA will prevent the function of small GTPases that, in turn, enhances the expression of various BMPs. BMPs are essential for neuronal growth, proliferation, and differentiation. Thus, EPA and DHA modulate brain growth and development, and neuronal differentiation. This action is in addition to their (EPA and DHA) ability to form an important constituent of neuronal cell membranes and involvement in memory formation and consolidation [24-26], explaining the beneficial action of EPA and DHA in the prevention and treatment of dementia and Alzheimer's disease [18-20,24-26,44,45]. In addition, the beneficial action of EPA and DHA in Alzheimer's disease, schizophrenia and dementia can also be attributed to the formation of anti-inflammatory compounds such as lipoxins, resolvins, protectins and maresins from EPA and DHA.

Based on these evidences, we propose that in transgenic *fat-1 *mouse will not only have increased amounts of EPA and DHA in the brain and other tissues and plasma but also show reduced activity of HMG-CoA reductase enzyme, altered SREBP-mediated gene transcription, decreased *Ras *activity and enhanced levels of various BMPs in the brain.

## Conclusions

**Figure 3:**
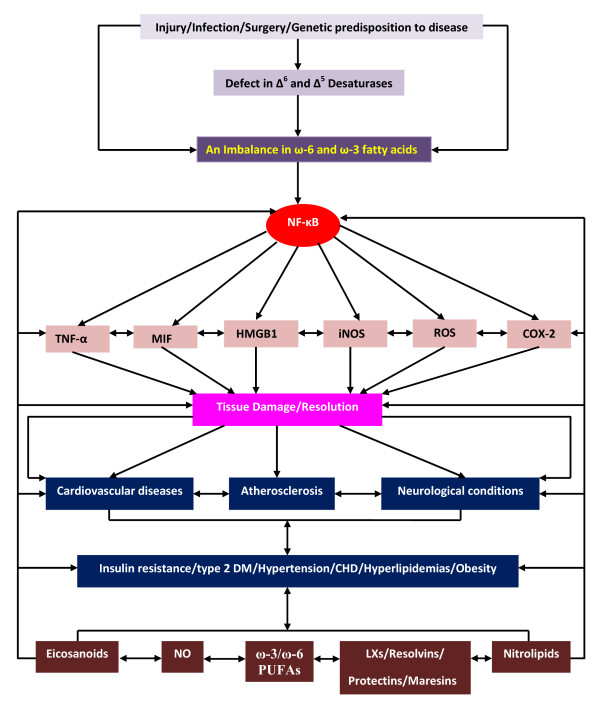
**Scheme showing the relationship among PUFAs and cardiovascular and neurological conditions**.

It is evident from the preceding discussion that EFAs and their metabolites including eicosanoids, LXs, resolvins, protectins and maresins and nitrolipids have many biological actions and are beneficial in the prevention of cardiovascular and neurological/psychiatric disorders. Since transgenic *fat-1 *mouse have increased concentrations of EPA and DHA in the brain and other tissues and plasma, we suggest that in this animal model the production of pro-inflammatory cytokines will be low; plasma and tissue concentrations of pro-inflammatory eicosanoids will be low with a simultaneous increase in the formation of anti-inflammatory compound such as lipoxins, protectins, resolvins and maresins; the cell membrane will be highly fluid with decreased HMG-CoA reductase activity and enhanced activity of eNOS; altered expression of genes for SREBPs; decreased *Ras *activity and high concentrations of various BMPs. In addition, we also propose that the expression of uncoupling protein-1 (UCP-1) in the vascular tissue and the expression of adhesion molecules (integrins) will be low and thus, the occurrence of atherosclerosis will be decreased in transgenic *fat-1 *mouse. In a recent study, we showed that in *fat-1 *transgenic mouse the expression of several genes concerned with inflammation, apoptosis, cell cycle, neurotransmitters, and hormones are altered [46] (see Tables 2 and 3). Thus, in transgenic *fat-1 *mouse there will be significant alterations in the concentrations and expression of cytokines, pro-inflammatory eicosanoids, insulin, neurotransmitters, PLA_2_, sphingosine kinase, caspases, farnesoid X receptor, PPARs, SREBPs, HMG-CoA reductase, transforming growth factor, cytokeratins, and nitric oxide synthase ([15,47-50] and see Table 4) that accounts for the decreased incidence of cardiovascular and neurological and psychiatric disorders (see Figure 3).

**Table 2:** Genes whose expression is altered in transgenic *fat-1 *mouse (see Ref. [46]).

Genes whose expression is down-regulated	Genes whose expression is up-regulated
Stearoyl-Coenzyme A desaturase 2 (Scd2)	Hydroxysteroid dehydrogenase-2, delta<5>-3-beta (Hsd3b2)

Prostaglandin D2 synthase (brain) (Ptgds)	Signal-induced proliferation-associated 1 like 1 (Sipa1l1)

Purkinje cell protein 4 (Pcp4)	Calpain 1 (Capn1)

Heat shock protein 2 (Hspb2)	Chloride channel calcium activated 6 (Clca6)

Apolipoprotein D (Apod)	Dopamine receptor D1 interacting protein (Drd1ip)

Sphingosine kinase 1 (Sphk1), transcript variant 2	Transforming growth factor, beta receptor II (Tgfbr2)

Low density lipoprotein receptor-related protein 1 (Lrp1)	3-hydroxy-3-methylglutaryl-Coenzyme A lyase (Hmgcl)

Lysophosphatidylglycerol acyltransferase 1 (Lpgat1)	

Phospholipase A2, group IVE (Pla2g4e) Dopamine receptor 4 (Drd4)	

DnaJ (Hsp40) homolog, subfamily C, member 5 (Dnajc5)	

Solute carrier family 32 (GABA vesicular transporter), (Slc32a1)	

Basal cell adhesion molecule (Bcam), mRNA	

Chemokine (C-C motif) receptor 5 (Ccr5), mRNA	

Peroxisomal biogenesis factor 11a (Pex11a)	

Heat shock protein 1, beta (Hspcb)	

Gamma-aminobutyric acid (GABA-A) receptor, beta 3 (Gabrb3)	

**Table 3:** Proteins that are either upregulated or downregulated in the brain of *fat-1 *transgenic mouse (see Ref. [46]).

Down-regulated proteins	Fold change
Caspase 4	-1.74

Connexin 32	-1.71

HSP70	-1.69

cytokeratin 19	-1.64

cytokeratin 8.12	-1.63

TRF1	-1.59

cdk6	-1.56

Desmin	-1.54

cytokeratin 13	-1.48

cytokeratin 7	-1.46

Calmodulin	-1.46

pan Cytokeratin	-1.45

Nedd8	-1.43

aCatenin	-1.43

NTF2	-1.38

Dystrophin	-1.38

**Up-regulated proteins**	**Fold change**

Phospolipase A2 group V	1.40

FAK Phospo (pY577)	1.45

Nicastrin	1.46

b-NOS	1.46

CRK-L	1.47

S-100	1.47

SGK	1.48

Caveolin1	1.49

Bcl-xl	1.50

ARTS	1.50

i-NOS	1.51

CAM Kinase IV	1.52

PTEN	1.52

PAR4	1.52

Neurofilament 200	1.54

Gamma Tubulin	1.56

MAP Kinase(Erk1)	1.56

Phospolipase c gamma 1	1.56

MAPK activated protein kinase-2	1.56

EGF receptor	1.58

MAP Kinase activated phospotyrosine	1.58

ERK5	1.58

Protein phosphatase 1	1.60

p35	1.60

PAK phospo (Ps212)	1.60

PKC alfa	1.62

PKD	1.62

Glutamate receptor NMDAR 2a	1.63

S-100 beta	1.63

Protein phosphatase 1	1.64

FAK Phospo (pS772)	1.65

DOPA Decarboxylase	1.66

PKC gamma	1.66

NFKB	1.67

JNK activated diphospo	1.69

Tau Phospho (pS199/202)	1.79

Synuclein monoclonal	1.79

Estrogen Receptor	1.81

GRB-2	1.95

MAP Kinase activated phospothreonine	1.98

**Table 4:** Proposed differences between the wild type and *Fat-1 *mouse.

Parameter	Wild type	*Fat-1 *mouse
Cell membrane Fluidity	↔	More fluid
Endothelial NO	↔	↑
IL-6, TNF-α, IL-1, IL-2,	↔	↓
MIF, HMGB1	↔	↓
IL-4, IL-10	↔	↑
HMG-CoA reductase activity	↔	↓
Plasma and tissue levels of EPA/DHA	↔	↑
Plasma and tissue levels of lipoxins, resolvins,	↓	↑
protectins and maresins	↓	↑
PGE_1_/PGI_2_/PGI_3_	↔	↑
*Ras *activity	↔	↓
BMPs	↔	↑
UCP-1	↔	↓
Expression of Adhesion Molecules	↔	↓
PPARs	↔	↑
Inflammatory diseases*	common	Uncommon/less severe
Blood pressure	Normal	↓
Type 1 and Type 2 diabetes	common	Uncommon/less severe
CHD	common	Uncommon/less severe

**CHD **= Coronary heart disease

Inflammatory diseases*include: rheumatological conditions such as rheumatoid arthritis, lupus, scleroderma, ankylosing spondylitis, vasculitis, interstitial lung disease, etc; neurological conditions such as stroke, Huntington's disease, Alzheimer's disease, depression, schizophrenia, familial and non-familial neurodegenerative conditions, amyotrophic lateral sclerosis, bulbar palsy, pseudobulbar palsy, inflammatory diseases of the bowel such as ulcerative colitis, Crohn's disease, Celiac disease, etc., psoriasis, glomerulonephritis, atherosclerosis, Parkinson's disease, hepatitis both specific and non-specific types, non-alcoholic fatty liver disease, insulin resistance, diabetes mellitus, metabolic syndrome, osteoporosis and all other conditions in which inflammation plays a role.

↔ Indicates normal frequency or incidence of the disease.

In the *Fat-1 *mouse, all the diseases enumerated above either will be less common or when they are induced or occur will run a much milder course compared to the severity of the disease seen in the wild type mouse.

In addition, recent studies showed that DHA yields an array of potentially bioactive lipid compounds such as cyclopentenone isoprostanes and cyclopentenone neuroprostanes (A(4)/J(4)-NPs), which are highly reactive and similar in structure to anti-inflammatory cyclopentenone prostaglandins. The synthetic A(4)/J(4)-NP, 14-A(4)-NP (A(4)-NP), potently suppressed lipopolysaccharide-induced expression of inducible nitric-oxide synthase, cyclooxygenase-2, NF-kappaB activation via inhibition of Ikappa kinase-mediated phosphorylation of IkappaBalpha in macrophages. The effects of A(4)-NP were found to be independent of peroxisome proliferator-activated receptor-γ (PPAR-γ) and were dependent on an intact reactive cyclopentenone ring. Paradoxically, free radical-mediated oxidation of DHA greatly enhanced its anti-inflammatory potency, an effect that closely parallels the formation of A(4)/J(4)-NPs. Furthermore, chemical reduction or conjugation to glutathione, both of which eliminate the bioactivity of A(4)-NP, also abrogated the anti-inflammatory effects of oxidized DHA. Thus, A(4)/J(4)-NPs, formed via the oxidation of DHA, are potent inhibitors of NF-kappaB signaling and contribute to the anti-inflammatory actions of DHA [51,52], suggesting that the anti-inflammatory properties of ω-3 fatty acids are closely associated and dependent on the novel interactions between lipid peroxidation products and inflammation. It is interesting to note that cyclopentenone isoprostanes (IsoPs), highly reactive structural isomers of the bioactive cyclopentenone prostaglandins PGA_2 _and PGJ_2_, are formed non-enzymatically as products of oxidative stress in vivo and 15-A_2_-IsoPs induced oxidative stress to inhibit the NF-kappaB pathway at least partially via a redox-dependent mechanism. 15-J_2_-IsoP induced RAW cell apoptosis again via a PPAR-γ-independent mechanism. These findings suggest that oxidative stress is beneficial at times by inducing the formation of cyclopentenone IsoPs that may serve as negative feedback regulators of inflammation [52,53].

It may be mentioned here that the beneficial actions of DHA and EPA in neurological conditions described above is not without controversy. For instance, Bate et al [54,55] reported that pre-treatment with DHA or EPA significantly reduced the survival of cortical or cerebellar neurons incubated with HuPrP82-146, a peptide derived from the prion protein, or with Abeta 1-42, a peptide found in Alzheimer's disease. They noted that treatment with DHA or EPA reduced the free cholesterol content of neuronal membranes that increased the kinetics of incorporation. In untreated neurons, FITC-HuPrP82-146 migrated to caveolin-1 containing lipid rafts, triggered the migration of cytoplasmic phospholipase A_2 _(cPLA_2_) into caveolin-1 containing rafts, and increased prostaglandin E_2 _production. They also observed that the activation of cPLA_2 _and prostaglandin E_2 _production were both increased in neurons pre-treated with DHA, suggesting that DHA or EPA alter cell membrane fluidity that could result in increased amounts of HuPrP82-146 localizing to caveolin-1 containing rafts, increased activation of cPLA2, prostaglandin E_2 _production, caspase-3 activity and reduced neuronal survival. These observations indicate that under some specific conditions ω-3 fatty acids EPA and DHA may actually accelerate neuronal loss in the terminal stages of prion or Alzheimer's diseases. This study suggests that further studies are needed to understand the close interaction between ω-3 fatty acids and free radicals and the series of products formed and the exact sequence of formation of various anti- and pro-inflammatory products that ultimately determine the neuronal loss or their growth. It is possible that under low oxidative stress conditions beneficial products such as lipoxins, resolvins, protectins, maresins, isoprostanes and A(4)/J(4)-NPs are formed and under excessive oxidative stress conditions cPLA_2 _is activated leading to the formation of PGE_2 _leading to neuronal loss (see Figure 2).

## Competing interests

The authors declare that they have no competing interests.

## Authors' contributions

Some of the experiments described in the present study were performed in the laboratory of LGP. Both the authors drafted and approved the final manuscript.
